# ATF3 deficiency impairs the proliferative–secretory phase transition and decidualization in RIF patients

**DOI:** 10.1038/s41419-021-03679-8

**Published:** 2021-04-12

**Authors:** Zhilong Wang, Yang Liu, Jingyu Liu, Na Kong, Yue Jiang, Ruiwei Jiang, Xin Zhen, Jidong Zhou, Chaojun Li, Haixiang Sun, Guijun Yan

**Affiliations:** 1 Reproductive Medicine Center, The Affiliated Drum Tower Hospital of Nanjing University Medical School, 210008 Nanjing, People’s Republic of China; 2 Nanjing University Medical School, 210008 Nanjing, People’s Republic of China

**Keywords:** Infertility, Medical research

## Abstract

Decidualization is a complex process involving cellular proliferation and differentiation of the endometrial stroma and is required to establish and support pregnancy. Dysregulated decidualization has been reported to be a critical cause of recurrent implantation failure (RIF). In this study, we found that Activating transcription factor 3 (ATF3) expression was significantly downregulated in the endometrium of RIF patients. Knockdown of ATF3 in human endometrium stromal cells (hESCs) hampers decidualization, while overexpression could trigger the expression of decidual marker genes, and ameliorate the decidualization of hESCs from RIF patients. Mechanistically, ATF3 promotes decidualization by upregulating FOXO1 via suppressing miR-135b expression. In addition, the endometrium of RIF patients was hyperproliferative, while overexpression of ATF3 inhibited the proliferation of hESCs through CDKN1A. These data demonstrate the critical roles of endometrial ATF3 in regulating decidualization and proliferation, and dysregulation of ATF3 in the endometrium may be a novel cause of RIF and therefore represent a potential therapeutic target for RIF.

## Introduction

Reciprocal intimate cross-talk between the activated blastocyst and the receptive uterus is essential for successful implantation, and therefore, for pregnancy outcome^1^. The desire to have children is powerful and widespread, however, about 15% of couples are childless because of infertility, which is a worldwide social and economic concern^2^. Currently, due to impressive advances in assisted reproductive technology (ART) and, in particular, the advent of more effective embryo selection and cryopreservation, many underlying causes of human infertility have been overcome; however, there are still many patients who continue to experience ART failure after serial IVF attempts^3^, probably owing to embryos being transferred into a nonreceptive uterus. Recurrent implantation failure (RIF) is one of the major causes of infertility in ART programs, and these pregnancy failures are believed to be mainly due to defects in early pregnancy events, including implantation and decidualization^4^.

In recent years, it has become apparent that endometrial factors have an important role in implantation^5,6^. The human endometrial cycle can be divided into 2 phases: the proliferative phase, which is marked by the active growth of stromal, epithelial, and vascular cells, and the secretory phase, during which the endometrium undergoes decidualization to prepare for implantation^7,8^. The proper transition between the proliferative and secretory phases is a prerequisite for the establishment of the WOI and the subsequent pregnancy. Decidualization deficiency or abnormality may be one of the major maternal causes of RIF^9,10^. Besides, the receptivity window of RIF patients was delayed by 1–3 days^11,12^, suggesting that the acquisition of the endometrial receptivity is slower in RIF patients. Nevertheless, the critical underlying molecular mechanisms remain largely unknown.

Activating transcription factor 3 (ATF3) belongs to the ATF/CREB transcription factor family, which binds to the consensus ATF/cAMP response element in numerous promoters^13,14^. It can also regulate various biological functions independent of its transcriptional activity^15,16^. ATF3 acts as a hub of the cellular adaptive-response network and has vital roles in modulating metabolism, immunity, the cell cycle, and differentiation. Distinct from other ATF/CREB proteins, ATF3 expression is rapidly induced by a wide range of extracellular signals, such as nutrient deprivation, endoplasmic reticulum stress, cytokines, chemokines, and hormones^17–19^. However, little is known about whether uterine ATF3 has any role in decidualization, the proliferative–secretory phase transition of the menstrual cycle, and the pathological relationship with RIF. In this study, we describe a novel mechanism of dysregulation of ATF3 in the endometrium from RIF patients hampers decidualization that may be a new cause of RIF.

## Results

### ATF3 is deficient in the endometrium of RIF patients

**Fig. 1:**
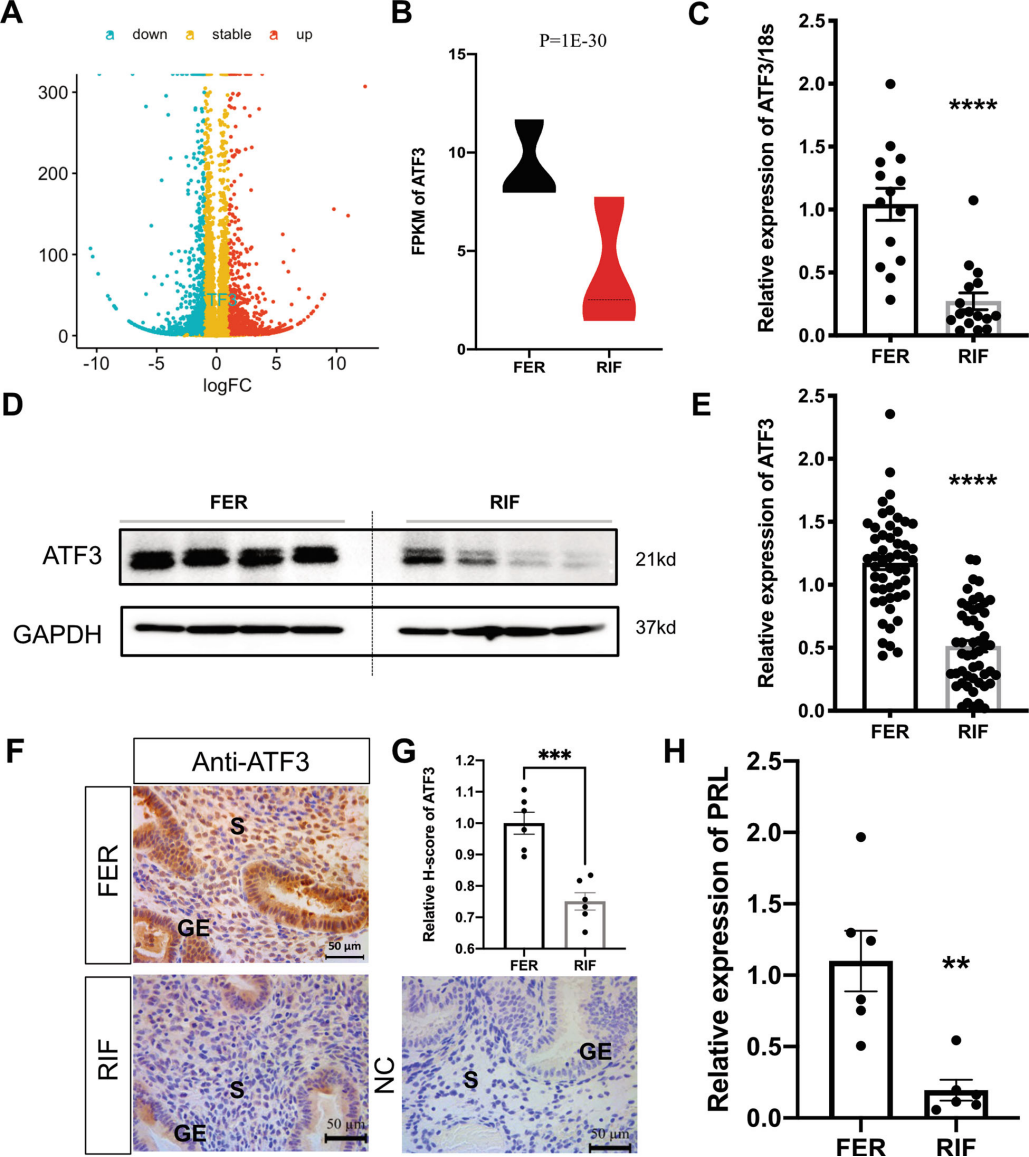
Aberrant expression of ATF3 in the endometrium of RIF patients. **A**, **B** RNA-seq results showed that ATF3 in the endometrium of RIF patients was downregulated (RIF) compared with that in fertile controls (FER). **C** ATF3 expression in RIF and FER endometrium was measured by quantitative PCR (qPCR). The expression of ATF3 was normalized to 18S rRNA expression, and the results are presented relative to FER stage (*n* = 14 for FER group and *n* = 16 for RIF group). ****P < 0.0001 compared with the FER group. **D**, **E** Immunoblotting for ATF3 in the endometrium of RIF patients and the FER group. Quantitative densitometry analysis indicated that ATF3 levels were downregulated in RIF patients (n = 51). ****P < 0.0001 compared with the FER group. **F**, **G** Representative immunohistochemical images depicting the expression of ATF3 in the endometrium from fertile women and RIF patients. The negative control (NC) was nonspecific rabbit serum. The *H*-score of ATF3 expression in the endometrial stromal cells was calculated with IHC-Profiler and ImageJ (scale bar = 50 µm, *n* = 6, ***P < 0.001). **H** qPCR was performed to detect the expression of PRL in the endometrium of RIF patients, normalized to 18S rRNA expression (*n* = 6).

In our previous study, we demonstrated that the endometrium of RIF patients displays a distinct transcriptional profile and suggests that dysregulation of decidualization may be a cause of implantation failure^10^. Through RNA-seq analysis, we found that ATF3 expression was significantly downregulated in the endometrium of RIF women (Fig. 1A, B), while other members of ATF/CREB did not change significantly (Supplemental Fig. S1). qPCR was then used to validate that in the mid-secretory phase, subjects with RIF had a 0.5-fold lower ATF3 expression level than controls (Fig. 1C), consistent with the western blot results (Fig. 1D, E). As shown in Fig. 1F, G, the expression of ATF3 in the endometrial stromal cells from RIF patients was significantly decreased, as determined by immunohistochemical staining. Through analysis of publicly accessible data banks, we found that the placenta exhibits a high level of ATF3 expression, whereas its level ranges from medium to high in the uterus and endometrium, indicating an important role for ATF3 in human endometrial cycling (Supplemental Fig. S1). We, therefore, wondered whether an element of the infertility phenotype could be caused by an alteration in the expression of ATF3. In addition, we found that the expression of the decidualization marker PRL was decreased in the endometrium from RIF patients (Fig. 1H).

### Spatiotemporal expression of ATF3 in the human endometrium

**Fig. 2:**
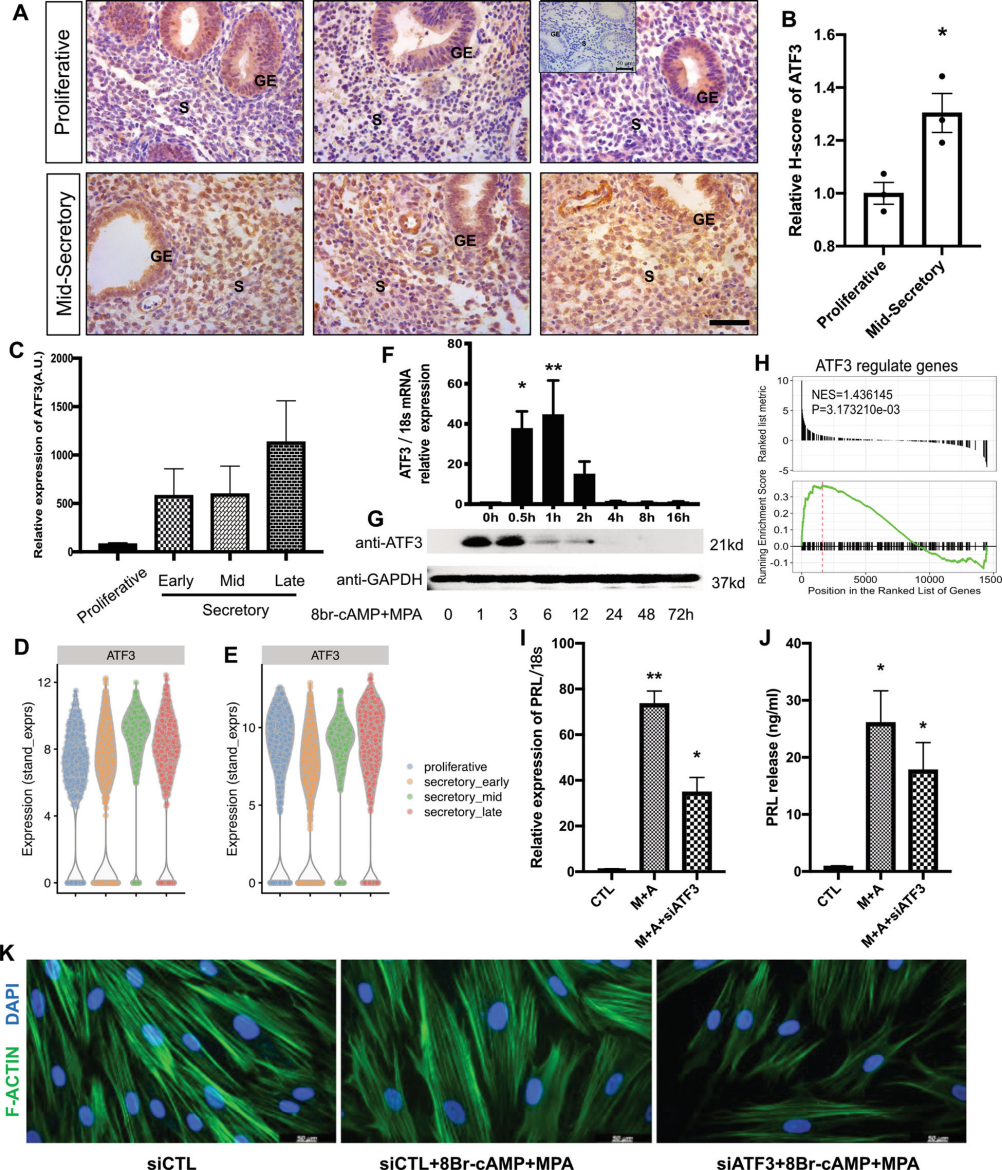
The temporal expression and function of ATF3 in the endometrium. **A**, **B** Immunohistochemistry analysis with an anti-ATF3 antibody. Proliferative and mid-secretory phase endometrial tissue samples from normal fertile women. The negative control (NC) was nonspecific rabbit serum. The *H*-score of ATF3 expression in the endometrial stromal cells was calculated with IHC-Profiler and ImageJ (Scale bar = 50 µm, *n* = 3, *P < 0.05). **C** Expression of ATF3 in proliferative and early-, mid-, and late-luteal phase endometrium. Each bar represents an individual biopsy. The data were retrieved from microarray data deposited in the Gene Expression Omnibus (GDS2052). **D**, **E** ATF3 expression pattern analyzed with a recent single-cell RNA-seq analysis in stromal cells (**D**) and all cells (**E**). The data were retrieved from microarray data deposited in the Gene Expression Omnibus (GSE111976). **F** The expression pattern of ATF3 in hESCs treated with 0.5 mM 8-Br-cAMP and 1 μM MPA (M + A) for different periods of time (0, 0.5, 1, 2, 4, 8, or 16 h) was evaluated by qPCR. *P < 0.05, **P < 0.01. **G** The expression pattern of ATF3 in hESCs treated with 0.5 mM 8-Br-cAMP and 1 μM MPA (M + A) for different periods of time (0, 1, 3, 6, 12, 24, 48, or 72 h) was evaluated by western blot. **H** GSEA results showed that the ATF3-regulated gene term was enriched during in vitro decidualization. **I**, **J** hESCs were transfected with siATF3 or siCtl for 48 h and then treated with a decidualization stimulus. The expression and secretion of PRL were measured by qPCR and ELISA, respectively. *P < 0.05, **P < 0.01 compared with the CTL/M + A group (8-Br-cAMP+MPA). **K** Immunofluorescence was performed to analyze the morphological transformation of hESCs.

To address the pathophysiological significance of ATF3 during early pregnancy, we then analyzed the ATF3 expression pattern in the human endometrium by immunohistochemical staining. The results show that the ATF3 protein abundance was higher in the mid-secretory phase than in the proliferative phase of the endometrium, especially in the endometrial stromal cells (Fig. 2A, B). We also analyzed the relative expression of temporal ATF3 expression across the cycle from a public dataset (Fig. 2C). Additionally, a recent single-cell RNA-seq analysis suggests that ATF3 may be a probable driver for decidualization^20^, and using this data we found that ATF3 is elevated in the stromal cells since the early-secretory phase (Fig. 2D). Besides, the expression of ATF3 in all cells of the endometrium is upregulated in the mid-secretory compared with the early-secretory phase (Fig. 2E).

We also examined the levels of ATF3 and other members of the ATF/CREB family in hESCs treated with 8-Br-cAMP plus MPA for 0.5–16 h using qPCR. The results reveal that ATF3 was rapidly elevated by ~40-fold at ~1 h in hESCs (Fig. 2F), while other ATFs showed no significant change (Supplemental Fig. S2). Western blot analysis also confirmed that the expression of ATF3 was increased after in vitro decidualization (Fig. 2G). Gene set enrichment analysis (GSEA) results showed that the ATF3 target term was significantly upregulated during decidualization (NES = 1.436, *P* < 0.01) (Fig. 2H). The results indicate that decidualization stimuli could upregulate the expression of ATF3 in the stroma of the endometrium.

### Lack of ATF3 impairs decidualization

Various functions have been ascribed to decidual prolactin (PRL) which is now established as a widely used marker to assess the differentiation status of hESCs in culture. As shown in Supplemental Fig. S3A, the expression of ATF3 showed a positive relationship with PRL in the endometrium. When hESCs were transfected with small interfering RNA specific for ATF3 for 48 h followed by treatment with 8-Br-cAMP and MPA for 3 days, the expression and secretion of PRL were substantially decreased (Fig. 2I, J). We further examined the effect of ATF3 on the reorganization of the cytoskeleton, which reflects a morphological marker of decidualization^21^. As shown in Fig. 2K, decidualized hESCs displayed polygonal cell morphologies with a random distribution of F-actin filaments. When knockdown of endogenous ATF3, stromal cells maintained a fibroblast-like phenotype under decidualization stimuli. Furthermore, adenovirus-mediated overexpression of ATF3 in hESCs markedly increased the expression and secretion of PRL in the absence of exogenous hormones (Supplemental Fig. S3B, C). In addition, ATF3 overexpression resulted in a noticeable transformation from a long fibroblast-like shape into a rounder shape, and the actin filaments were randomly arranged (Supplemental Fig. S3D). Together, these results suggest that ATF3 may be functionally important for the regulation of decidualization.

### ATF3 increases PRL through transcriptionally independent regulation of FOXO1

**Fig. 3:**
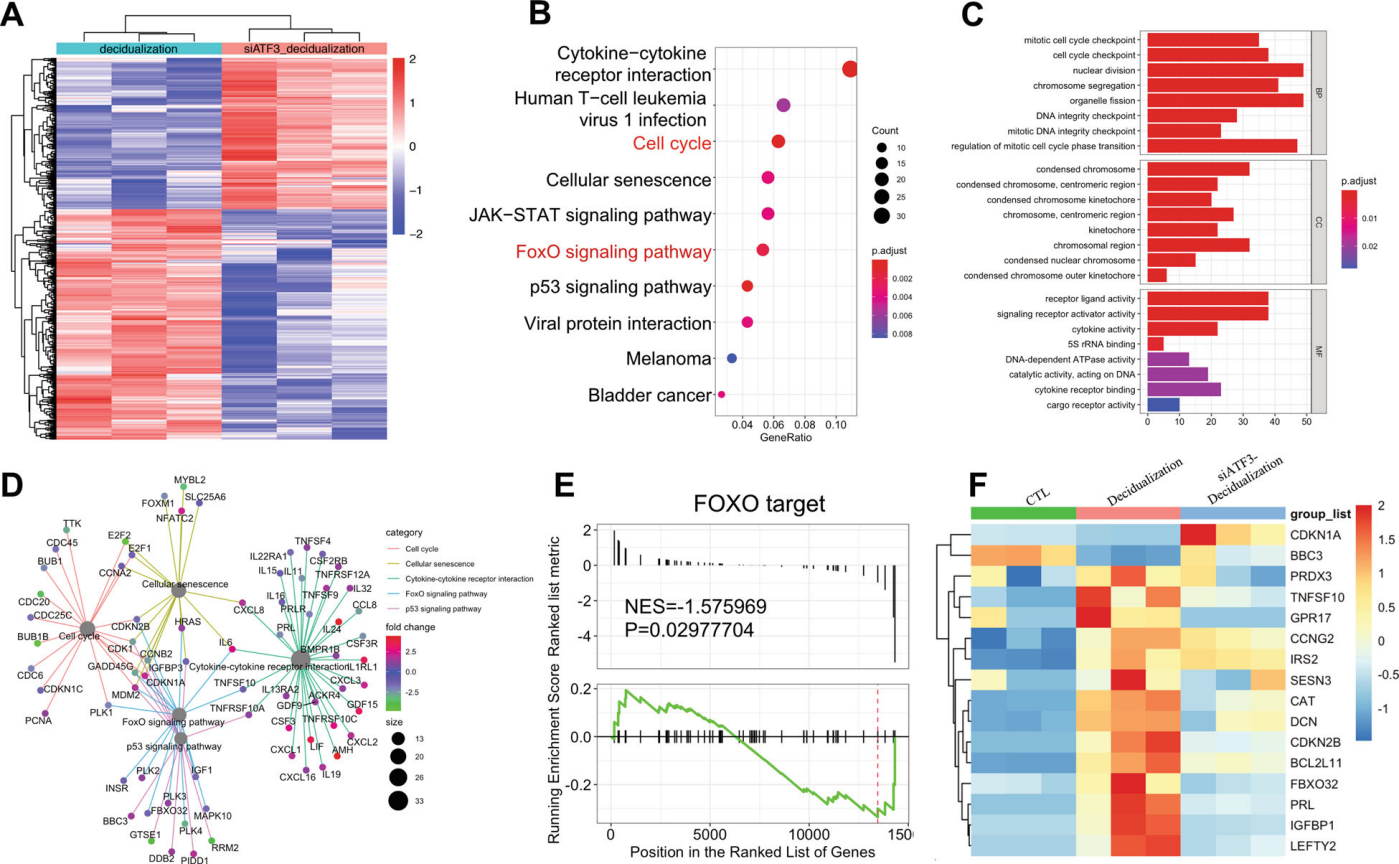
DEGs in hESCs after knockdown of ATF3 during in vitro decidualization. **A** Heat map showing hierarchical clustering of gene expression in decidualization and knockdown of the ATF3 group. **B**, **C** KEGG pathway enrichment analysis and GO analysis of the DEGs. **D** The DEGs related to the altered KEGG pathways. **E** GSEA results showed that the FoxO-targeted gene term was downregulated when ATF3 was silenced. **F** The relative expression of FoxO1 target genes, such as PRL, IGFBP-1, DCN, and LEFTY2.

RNA-seq analysis was performed to identify genes that are regulated by ATF3 during in vitro decidualization. As illustrated in Fig. 3A, there were 689 differentially expressed genes with 274 upregulated and 415 downregulated when ATF3 was silenced compared with those in the siCTL group during decidualization. In addition, decidualization marker genes, such as PRL, IGFBP-1, and LEFTY2, were significantly downregulated in the siATF3 group (Fig. 3D, F). To explore the mechanism by which ATF3 regulates decidualization, we further performed KEGG enrichment analysis based on the differentially expressed genes (DEGs). Interestingly, as one of the pathways that is affected most by ATF3, the cell cycle and FoxO signaling pathways were suggested to have a significant role (Fig. 3B, C, E, F).

**Fig. 4:**
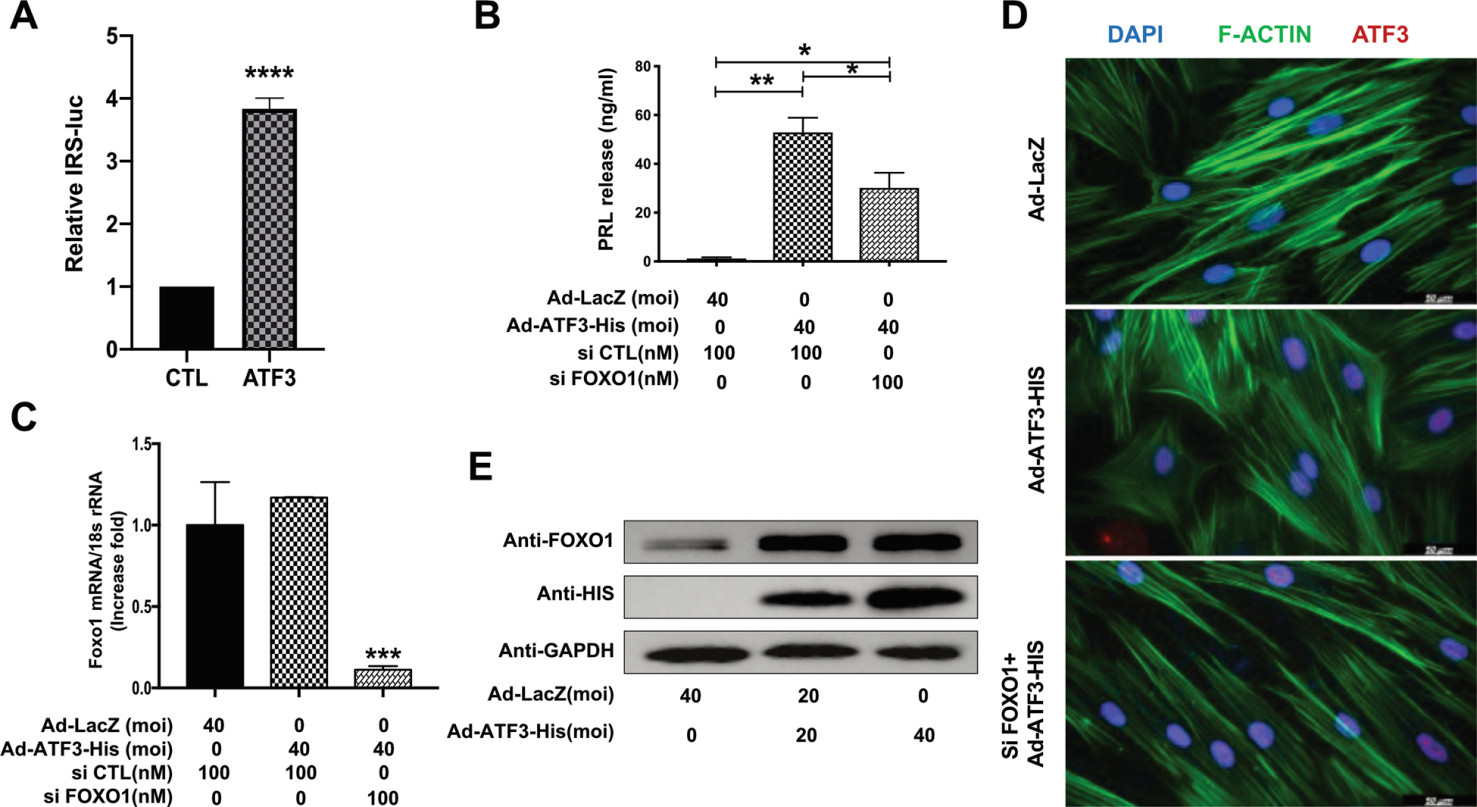
ATF3 regulates decidualization mediated by FOXO1. **A** hESCs were treated with Ad-LacZ or Ad-ATF3-His for 48 h and transfected with IRS-luc and Rinella for dual-luciferase assays. ****P < 0.0001. **B**–**D** hESCs were transfected with Ad-ATF3-his (0 or 40 MOI) or Ad-LacZ. si-FOXO1 was used to knockdown the expression of FOXO1, and prolactin secretion was measured by an enzyme-linked fluorescent assay (ELFA). *P < 0.05. Immunofluorescence was performed to analyze the morphological transformation of hESCs. Scale bar = 50 μm. **E** hESCs were transfected with Ad-ATF3-his (0, 20, or 40 MOI) or Ad-LacZ. FOXO1 and His protein expression levels were measured by western blot.

We then performed dual-luciferase reporter assays in hESCs and found that overexpression of ATF3 caused a significant increase in the transcriptional activity of FOXO1 (IRS-luc) (Fig. 4A). FOXO1 is important for the induction of differentiation markers, such as PRL, IGFBP-1, and LEFTY2, in decidual cells^22^. Therefore, we postulated that FOXO1 contributes to the ATF3-induced expression of PRL. As shown in Fig. 4B, C, overexpression of ATF3 increased PRL secretion, which was clearly attenuated by FOXO1 knockdown. Knockdown of FOXO1 with siRNA efficiently inhibited cytoskeletal rearrangement (Fig. 4D). Thus, upregulation of PRL expression by ATF3 may be specifically mediated by regulating FOXO1.

Then, we investigated the effect of ATF3 on FOXO1 and found that compared with the control group, adenovirus-mediated overexpression of ATF3 in hESCs resulted in a concentration-dependent increase in FOXO1 protein expression, as determined by western blot analysis (Fig. 4E). However, the mRNA level of FOXO1 showed no significant difference in hESCs with ATF3 knockdown or overexpression (Fig. 4B and Supplemental Fig. S4C). These findings suggest that ATF3 may upregulate the expression of FOXO1 in a transcription-independent manner.

### miR-135b mediates the regulation of FOXO1 by ATF3 during decidualization

**Fig. 5:**
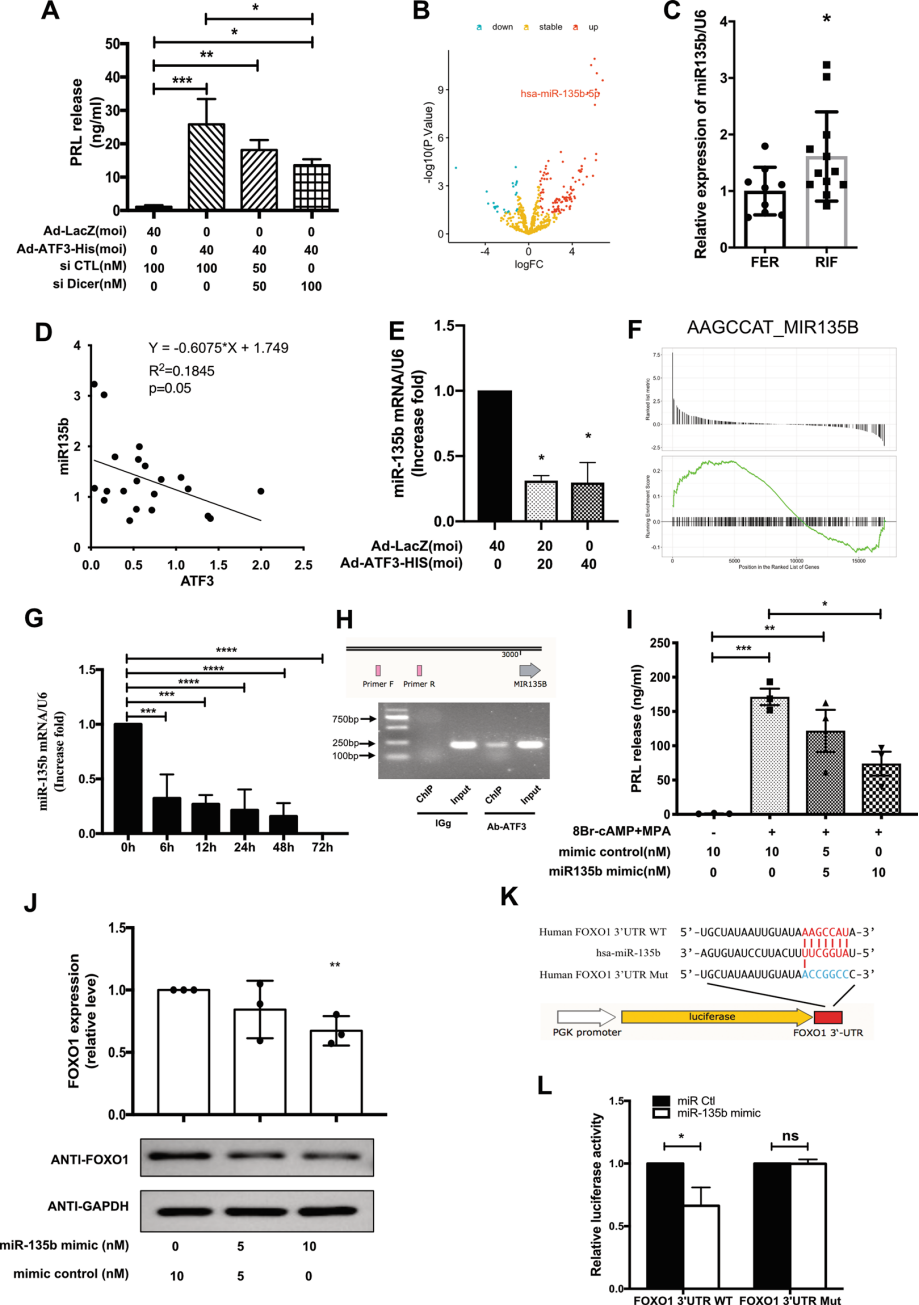
miR-135b mediates the regulation of FOXO1 by ATF3 during decidualization. **A** hESCs were transfected with Ad-ATF3-his (0 or 40 MOI) or Ad-LacZ. si-Dicer was used to knockdown the expression of Dicer, and prolactin secretion was measured by ELFA. *P < 0.05, **P < 0.01, ***P < 0.001. **B**, **C** miRNA array and qPCR were performed to detect the differentially expressed miRNAs in RIF patients. Data were downloaded from the GEO database (GSE71332). The expression of miR-135b was validated by qPCR and normalized to U6 expression (*n* = 9 for FER group and 12 for RIF group, *P < 0.05). **D** The correlation of ATF3 expression and miR-135b with the data in **B**. **E** hESCs were transfected with Ad-ATF3-his (0, 20, or 40 MOI) or Ad-LacZ. miR-135b expression levels were analyzed by qPCR. *P < 0.05. **F** GSEA results showed that the AAGCCAT miR-135B term was activated after overexpression of ATF3. **G** hESCs were treated with 8-Br-cAMP+MPA for different times, and then the expression of miR-135b was measured by qPCR. ***P < 0.001, ****P < 0.0001. **H** ChIP-PCR was used to detect the binding site of ATF3 on the promotor of miR-135b. **I** hESCs were transfected with miR-135b mimic (10 nM) or mimic control. After 24 h, the cells were treated with 0.5 mM 8-Br-cAMP and 1 μM MPA for an additional 3 days. Prolactin released into the medium was measured by ELFA. *P < 0.05, **P < 0.01, ***P < 0.001. **J** hESCs were transfected with hsa-miR-135b mimic (0, 5, or 10 nM) or mimic control. FOXO1 protein levels were analyzed by western blot. **P < 0.01. **K**, **L** Putative 7 bp paired miR-135b/a-target sites in the 3′UTR of human FOXO1 mRNA. Analysis of miR-135b modulation of the wild-type and mutant FOXO1 3′ UTR luciferase reporter activity. *P < 0.05, ns not significant.

As shown in Fig. 3B, we also found that overexpression of ATF3 significantly changed the microRNAs in the cancer pathway. Emerging evidence suggests that microRNAs may have a role in decidualization and implantation^23,24^. To investigate whether there is a microRNA involved in ATF3-induced decidualization, we introduced si-Dicer to ATF3-overexpressing cells and found that si-Dicer attenuated decidualization in cells treated with ATF3 (Fig. 5A). Differential microRNA expression was found between RIF women and fertile women^25^. Through analyzing miRNA-mRNA network, we found that miR-135b may be a potent regulator of FOXO1. In addition, the hsa-miR-135b mRNA levels in RIF patients were upregulated compared to those in fertile women (Fig. 5B, C).

We further found a moderately negative correlation of the expression of ATF3 and miR-135b in mid-secretory endometrial samples from women with RIF and fertile controls (*r* = 0.4389, *P* = 0.0465) (Fig. 5D). qPCR and GSEA results showed that ATF3 downregulated miR-135b in hESCs (Fig. 5E, F), and ChIP-PCR was performed to confirm that ATF3 could bind to the upstream promoter of miR-135 (Fig. 5H). We constructed a pair of pmirGLO dual-luciferase miRNA target expression vectors that contained the wild-type (FOXO1 3′UTR WT) and mutant target site (FOXO1 3′UTR Mut) for the 3′UTR of FOXO1 separately (Fig. 5K) and found that miR-135b significantly suppressed FOXO1 translational efficiency in hESCs (Fig. 5L). The expression of miR-135b in hESCs was rapidly downregulated in a time-dependent manner when treated with 8-Br-cAMP and MPA, as determined by quantitative real-time PCR analysis (Fig. 5G). And miR-135b downregulated the protein expression of FOXO1 in hESCs, as determined by western blot analysis (Fig. 5J). Furthermore, miR-135b significantly attenuated decidualization after treated with 8-Br-cAMP and MPA (Fig. 5I).

### ATF3 regulates the proliferative–secretory transition and thus participates in controlling the WOI in RIF patients

**Fig. 6:**
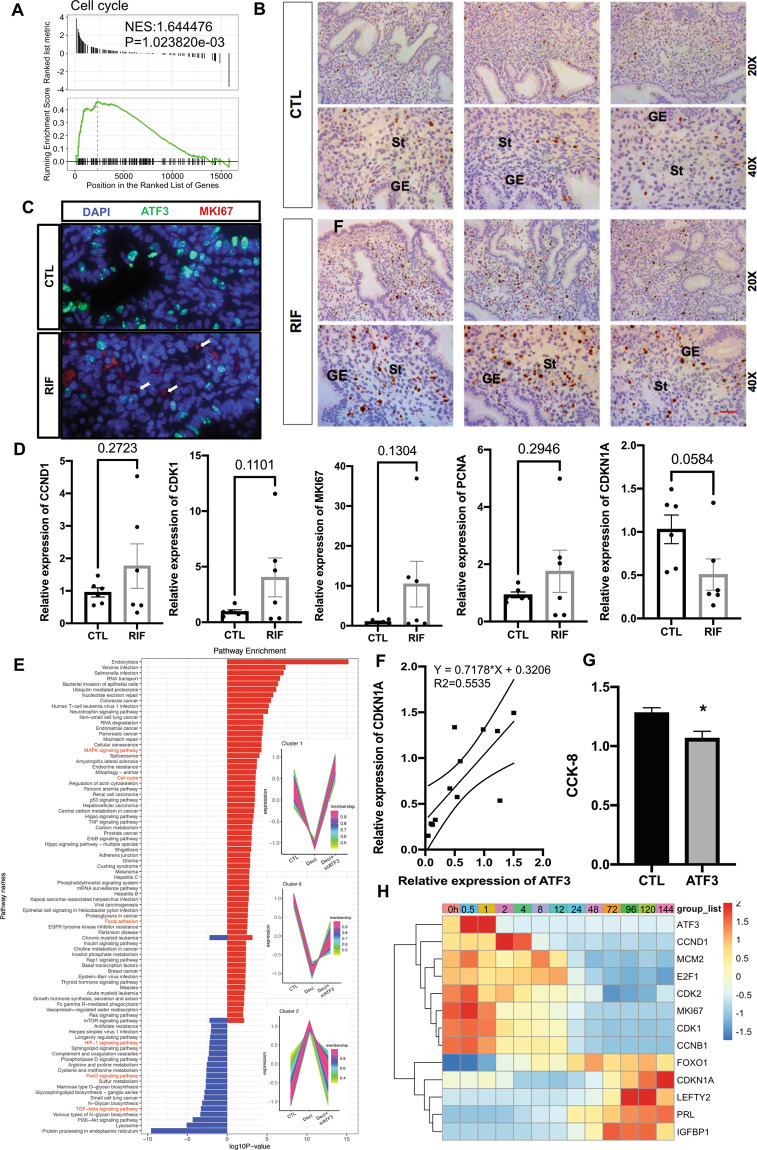
ATF3 regulates the cell cycle of hESCs. **A** GSEA results showed that the cell cycle term was activated in the endometrium of RIF patients (NES: 1.644476, *P* = 1.023820e−03). **B**. MKI67 staining was used to measure the proliferation of stromal cells in the endometrium of RIF patients and fertile controls (ST, stromal cells, GE, glandular epithelium, bar = 50 µm, *n* = 5). **C** immunofluorescence was performed to determine the expression of ATF3 and MKI67 in endometrium from fertile controls and RIF patients. **D** The cell cycle-regulated genes were measured by qPCR in the endometrium of RIF patients and fertile controls, *n* = 6. **E** TC-seq analysis was performed to analyze the DEGs in the control, decidualization, and siATF3+decidualization groups. Genes in clusters 1 and 6 were upregulated by siATF3, while those in cluster 2 were downregulated. **F** The correlation of ATF3 expression and CDKN1A. **G** hESCs were treated with Ad-ATF3-His or Ad-LacZ, and a CCK-8 assay was performed to detect the cell proliferation rate. **H** Genes related to cell proliferation and decidualization change regularly during in vitro-induced decidualization.

As mentioned above, DEGs regulated by ATF3 were related to the cell cycle pathway. We analyzed the gene profiles in RIF patients and found that endometrial stromal cell proliferation in RIF patients was hyperactive (Fig. 6A). Immunobiological staining showed that there were more MKI67-positive cells in RIF patients than in controls (Fig. 6B). The expression of CCND1, CDK1, MKI67, PCNA were upregulated, while CDKN1A in endometrium from RIF patients were relatively lower than fertile controls as determined by qPCR (Fig. 6D), and the expression of ATF3 showed a positive relationship with CDKN1A (Fig. 6F). However, during decidualization, the cell cycle was inhibited in stromal cells (NES: −1.811620, *P* = 5.303467e−05) which is consistent with the results of ATF3-regulated genes, and overexpression of ATF3 in hESCs caused restrained proliferative activity (Fig. 6G). CDKN1A was downregulated, while CCND1, CDK2, and MKI67 were upregulated by overexpression of ATF3 (Supplemental Fig. S4D). As shown in Fig. 6E and Supplemental Fig. S4E, “TCseq” and “clusterProfiler” packages were used to analyze the genes expression and annotation in control, decidualization, and siATF3-decidualization groups, the results showed that a cluster of genes (cluster 2) that should be upregulated during decidualization was inhibited by siATF3, which were related to FoxO signaling, TGF-beta signaling, and HIF-1 signaling pathway, which are important for decidualization and/or implantation^26–28^. On the other hand, the genes (cluster 1, 6) downregulated during decidualization were upregulated by siATF3, which were enriched in cell cycle, MAPK signaling, and focal adhesion signaling pathway. In the mid-secretory phase endometrium, fewer MKI67-positive stromal cells in the endometrium from fertile control groups compared with RIF patients, and ATF3-positive stromal cells do not express of MKI67 (Fig. 6C). We then analyzed the dynamic genes expression during in vitro decidualization, ATF3 was elevated rapidly, and then genes related to cell proliferation were downregulated and the decidualization related genes were upregulated. (Fig. 6H). Consistent with that, ATF3 was upregulated very early in the secretory phase and decidualization in vitro (Fig. 2F, G); thus, ATF3 may act as a brake of proliferation and a trigger of decidualization during the establishment of the WOI. Since the receptivity window of RIF patients was delayed by 1–3 days, these results suggest that the hyperproliferation of endometrial stromal cells may disrupt the proliferative–secretory transition in RIF patients.

### Decidualization is augmented by overexpression of ATF3 in RIF patients

**Fig. 7:**
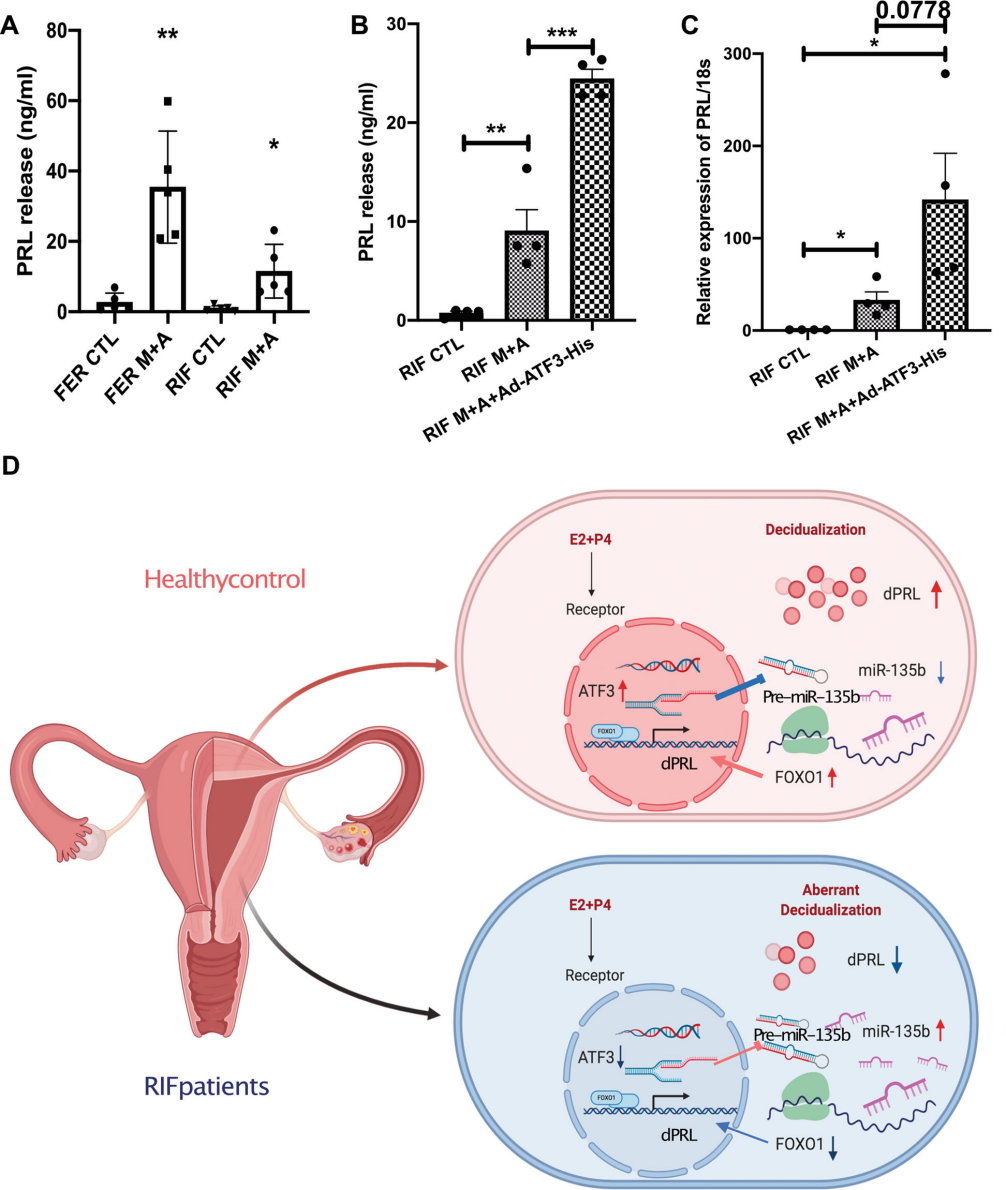
Decidualization is augmented by overexpression of ATF3 in RIF patients. **A** hESCs (from fertile controls and RIF patients) were treated with 8-Br-cAMP+MPA for 3 days. Prolactin secretion was measured by ELFA. *n* = 5, *P < 0.05, **P < 0.01. **B**, **C** hESCs from RIF patients were infected with Ad-ATF3-his (0 or 40 MOI) or Ad-LacZ for 48 h followed by treatment with 0.5 mM 8-Br-cAMP and 1 μM MPA for 3 days. Prolactin released into the medium was measured by ELFA and qPCR. *n* = 4, *P < 0.05, **P < 0.01. **D** Schematic representation of the role of ATF3 in the regulation of decidualization in RIF patients and fertile control groups.

The above results show that ATF3 had a key role in regulating decidualization and proliferation. We then investigated whether overexpression could augment decidualization in RIF patients. As shown in Fig. 7A, hESCs decidualization from RIF patients was dysregulated, as determined by significantly decreased decidual marker gene expression of PRL. Interestingly, overexpression of ATF3 increased PRL expression and secretion to a level nearly comparable with that of the fertile control (Fig. 7B, C).

We then investigated the mechanism of the augmentation on decidualization by overexpression of ATF3 through RNA-seq. Overexpression of ATF3 resulted in 547 DEGs, with 281 upregulated and 266 downregulated genes (Supplemental Fig. S4A). Overexpression of ATF3 highly regulated the cell cycle, estrogen signaling pathway, cAMP signaling pathway, cytokine–cytokine receptor signaling pathway, and microRNAs in cancer pathways (Supplemental Fig. S4B).

## Discussion

Despite the great progress that has been made in ART over the last 35 years, the embryo implantation rate is nevertheless still not satisfactory, and ~15% of patients undergoing ART procedures suffer from unexplained RIFs. Recently, a prospective interventional multicenter study found that the receptivity window of RIF patients was delayed by 1–3 days^12^, suggesting that the acquisition of the endometrial receptivity phenotype is slower in RIF patients than in healthy women. Here, we report that the proliferative–secretory transition was dysregulated in RIF patients and that ATF3 may be a key regulator.

Along with the discovery of new regulatory pathways that control steroid actions, an expectation grew that specific molecular defects in the endometrium would account for RIF or pregnancy loss^29,30^. Thus, there should be some under discovered mechanisms in the maternal uterus^31^. In our previous study, we found that ATF3 was located in both human endometrial epithelial cells and stromal cells, and its expression was dysregulated in RIF patients. In epithelial cells, ATF3 could promote embryo adhesion in vitro by transcriptionally increasing LIF expression^32^. In this study, we focused on the stromal cells to investigate the function of ATF3 on decidualization.

Decidualization is critical and essential for reproduction and involves a series of complex molecular and cellular events to prepare the endometrium for embryo implantation, and dysregulation of decidualization is one of the major causes of RIF^9,10^. We found that ATF3 was deficient in endometrial stromal cells from RIF patients, and knockdown of ATF3 impaired decidualization, as determined by dysregulated PRL expression and cytoskeletal reconstruction.

ATF3 is an adaptive-response gene that participates in various cellular processes to adapt to extra and/or intracellular changes, where it transduces signals from various receptors to activate or repress gene expression^33^. It has been reported that ATF3 overexpression impacted the proliferation, cell cycle, apoptosis, migration, and invasion of endometrial carcinoma, while estrogen and progesterone had a significant role in its pathogenesis^34^. Our previous study found that estrogen and progesterone could rapidly induce the expression of ATF3 in endometrial epithelial cells, and other studies have also suggested that ATF3 is a downstream gene of estrogen and progesterone^32,35,36^. Here, we found that the expression of ATF3 was increased in the early stage of in vitro decidualization and then decreased, while the expression of ATF3 in the endometrial tissue continued to increase until the mid to late-secretory phase of the menstrual cycle. This may because the 8-Br-cAMP+MPA-induced decidualization protocol could not completely simulate physiological conditions. Recent studies have found that PGE2-induced decidualization is a more physiological model than 8-Br-cAMP^37^, and that PGE2 has been reported to regulate the expression of ATF3^38^. It has been reported that impaired decidualization leads to altered stroma secretion, and disintegrating maternal-embryonic responses, predisposing patients to pregnancy loss^39,40^. PRL is a marker gene of decidualization, and knockout *Prl* leads to female infertility and lack of embryo implantation^41^. Besides, expression of endometrial prolactin during the “implantation window” was downregulated in patients affected by unexplained infertility and repeated miscarriages^42^. In this study, we found that endometrial stromal cells from RIF patients exhibit a reduced expression of PRL, while supplement with ATF3 could promote the secretion of PRL.

Endometrial decidualization involves the cytoskeletal transformation of stromal cells into secretory decidual cells, during which stromal fibroblasts transdifferentiate into large, epithelioid-like decidual cells. ATF3 is a highly conserved and multifunctional transcription factor that is known for its response to a range of stress signals, and it has been shown that ATF3 links the loss of epithelial polarity to defects in cell differentiation and cytoarchitecture^43^. Genomic and genetic approaches implicate ATF3 as a regulator of cytoskeletal organization and function. Yuan et al.^44^ reported that ATF3 could suppress the metastasis of bladder cancer by regulating gelsolin-mediated remodeling of the actin cytoskeleton. Indeed, we found that ATF3 is required for the cytoskeletal organization of endometrial stromal cells and that knockdown of this transcription factor significantly impaired the change in structural features and cell morphology during decidualization.

Currently, plenty of DEGs have been identified during decidualization, implicated in a broad variety of functions, such as the cell cycle, cytoskeletal remodeling, oxidative stress defense, FoxO signaling, modulation of transcription, and chemokine signaling^45^. When ATF3 was knockdown during decidualization, the cytokine–cytokine receptor interaction, FoxO signaling, HIF-1 signaling, and TGF-β signaling pathways were downregulated, while the cell cycle, MAPK signaling pathways were upregulated. In addition, overexpression of ATF3 enhances FOXO1 and its target gene expression in a posttranslational regulation. Emerging evidence suggests that microRNAs have a role in decidualization and implantation^15,16^. For example, miR-22, which is upregulated in women with RIF, could impair decidualization by suppressing the expression of Tiam1 and Rac1^46^. Through analyzing differentially expressed miRNAs in the endometrium of RIF patients, we found that miR-135b is a putative regulator of FOXO1, and ATF3 could downregulate the expression of miR-135b, thus promote the expression of FOXO1 indirectly.

Recently, clinical research found that the WOI of RIF patients may be delayed and that implantation failure could be linked to inadequate timing of embryo transfer^12^. In this study, we revealed that the endometrial stromal cells from RIF patients were hyperproliferative, while the cell cycle should be arrested during decidualization, suggesting that the transition from the proliferative to secretory phase was blocked. Furthermore, we found that silencing ATF3 during decidualization activated the cell cycle, while overexpression of ATF3 could limit the proliferation of hESCs. ATF3 is a regulator of the cell cycle in many other tissues or cell lines, e.g., ATF3 can inhibit the expression of AR, MMP2, and AKT, thereby inhibiting cell proliferation and invasion^47^. ATF3 expression is maintained at relatively low levels in quiescent cells. Extensive studies have characterized ATF3 as an adaptive-response gene that is induced by a wide variety of signals, including those initiated by cytokines or physiological stresses^33^. We also found that during the menstrual cycle, ATF3 was highly upregulated in the very early-secretory phase, at which point stromal cells stopped proliferating and began to differentiate. Therefore, we hypothesize that ATF3 may disrupt the cell cycle, trigger decidualization, and has a key role in the establishment of the WOI.

In summary, our study found that ATF3 has an important role in decidualization by upregulating FOXO1 via suppression of miR-135b expression and participates in the regulation of the proliferative–secretory phase transition by regulating the cell cycle. Overexpression of ATF3 could augment decidualization in RIF patients. These findings indicate that dysregulation of ATF3 in the endometrium is a novel cause of RIF and may therefore represent a potential therapeutic approach for RIF.

## Materials and methods

### Patients and consent to participate

The patients involved in this study were recruited from the IVF unit of the Reproductive Center of the Affiliated Drum Tower Hospital of Nanjing University Medical School from February 2014 to December 2019. A total of 51 RIF patients and 51 fertile patients were enrolled. Women with hydrosalpinx, endometriosis, or adenomyosis were excluded. The details of these patients are listed in Supplemental Table S1.

### Isolation and in vitro decidualization of hESCs

hESCs were isolated and cultured as previously described^48^. T-hESCs were obtained from ATCC. To induce decidualization, hESCs were cultured with final concentrations of 0.5 mM 8-Br-cAMP and 1 μM medroxyprogesterone acetate (MPA) (Sigma, St. Louis, MO) in DMEM/F12 (Gibco BRL/Invitrogen, Carlsbad, CA, USA) containing 2.5% charcoal/dextran-treated FBS (HyClone; Thermo Scientific, South Logan, UT, USA), 100 IU/ml penicillin, and 100 μg/ml streptomycin. We referred to this hormonal treatment as 8-Br-cAMP + MPA. Differentiation was assessed by examination of cell morphology under phase-contrast microscopy at various times during the treatment and by measuring the expression of decidualization-specific marker gene PRL as previously described^9^. For particular treatments, cells were pretreated with Ad-ATF3-His (abm, 071147A), Ad-LacZ, siATF3, or siCtl for 2 days and then treated with 8-Br-cAMP and MPA for 3 days.

### Immunofluorescence staining for ATF3 and F-actin filaments

hESCs grown in 24-well plates were treated with siRNA or Adenovirus for 2 days and then exposed to a decidualization stimulus of 8-Br-cAMP plus MPA for 3 days and then fixed with 4% paraformaldehyde (w/v) for 30 min at room temperature. Next, the cells were washed with PBS and permeabilized with 0.5% Triton X-100 in PBS at room temperature. Subsequently, the cells were blocked with 3% BSA in PBS and incubated with primary antibodies against ATF3 (1:50, 18655s, CST) at 4 °C overnight. Next, the cells were washed with PBST and then incubated with Donkey anti-Mouse IgG (H + L) Highly Cross-Adsorbed Secondary Antibody, Alexa Fluor 594 (1:100, A-21203, ThermoFisher Scientific), and fluorescein isothiocyanate-labeled phalloidin (1:300; P5282, Sigma, St. Louis, MO, USA) at room temperature. Cell nuclei were stained with DAPI.

### Dual-luciferase reporter assay

The wild type and mutant sequence of 3′ UTR of FOXO1 were cloned into the pmirGLO luciferase reporter plasmid (Promega) and sequenced to confirm the resulting plasmid by GenScript Biotech. hESCs were cultured in 24-well plates and transfected with the indicated plasmids. Cells were harvested, and the luciferase activities were analyzed after 48 h using a Dual-Luciferase Assay System (Promega) with a luminescence counter (Berthold Technologies) according to the manufacturer’s instructions. For normalization according to the transfection efficiency, firefly luciferase activity was normalized to the corresponding Renilla luciferase activity.

### RNA isolation and real-time PCR

Cells were lysed using TRIzol reagent (Sigma), and total RNA was extracted following the standard manufacturer’s protocol. Up to 2 µg of total RNA was reverse transcribed in a final volume of 20 µl to generate cDNA with 5× All-In-One RT Master Mix (abm, G492). Real-time PCR analysis was performed with SYBR Green dye and measured on an Analytik Jena instrument to quantify the ATF3, PRL, and FOXO1 mRNA levels. The housekeeping gene 18S rRNA was used as an internal control. The specific primer sequences are listed in Table S2.

### RNA-seq and data analysis

hESCs were cultured in 6 cm dishes and transfected with the indicated siRNA for 2 days, then decidualized for another 3 days. Total RNA of these cells was extracted with TRIzol reagent. The transcriptome sequencing and analysis were conducted by OE Biotech Co., Ltd. (Shanghai, China). Clean reads were mapped to reference genome (GRCh38.p12) using hisat2 and DEGs were identified using the DESeq (*P*-value < 0.05 and |log2foldChange| > 1 was set as the threshold for significantly differential expression). KEGG, GO and GSEA was performed using clusterProfiler^49^. And TCseq package^50^ was used to analyze the dynamic gene expression in the R software (R version 4.0.2).

### Western blot

Proteins were extracted as described previously^51^. Equal amounts (30 μg) of protein were separated on a 12% SDS-polyacrylamide gel and transferred onto polyvinylidene fluoride membranes (Millipore, Billerica, MA, USA). Immunoblotting was performed by incubating the membranes with primary antibodies against ATF3 (1:1000; HPA001562, Sigma), FOXO1 (1:2000; Cell Signaling Technology, Danvers, MA, United States), His-Tag (1:2000; M30111, Abmart, Shanghai, China), and GAPDH (1:10,000; AP0063, Bioworld Technology, MN, USA).

### Immunohistochemical staining

Fresh tissues were fixed, embedded in paraffin, and serially sectioned. Antigen retrieval was conducted by autoclaving the samples at 121 °C for 15 min in the presence of citrate antigen retrieval solution. The samples were incubated with antibodies against ATF3 (1:600; HPA001562, Sigma) overnight at 4 °C followed with immunohistochemical staining kits (Zhongshan Golden Bridge). Control sections were run concurrently with the experimental sections using nonspecific rabbit IgG. Nonspecific staining was not detected in the controls. H-score was calculated with IHC-Profiler^52^ and ImageJ software (imagej.nih.gov/ij) based on staining intensity and percentage of positive staining cells, and high positive staining scored 3, positive staining scored 2, low positive scored staining 1, and negative staining scored 0. In this study, we only calcite the staining score of the endometrium stromal cells.

### Chromatin immunoprecipitation (ChIP)/PCR assay

70% confluence hESCs were treated with Ad-ATF3-His or Ad-LacZ for 2 days. Crosslink, cell lysis, genomic DNA fragments extraction were performed as described^32^, and then immunoprecipitated using ATF3 antibody (ChIP Grade, ab207434, Abcam) and nonspecific IgG as technical control. The specific primers that were used to amplify the hsa-miR-135b promoter DNA fragments containing a putative ATF3 binding sequence were 5′-CAGCTGAAGCCCTCTTTCTG-3′ and 5′-AGGAGGGTCTGGGTAAAGGA-3′.

### Statistical analysis

All of the experiments were performed at least three times. Statistical analyses were performed using Prism version 7 software (GraphPad, LaJolla, CA) or R software. Data represent the mean ± SEM of biological replicates. Statistical differences in the mean expression values of the two treatment groups were compared using a two-tailed Student’s *t*-test. One-way ANOVA was performed for comparisons among more than two groups.

## Supplementary information

Supplemental Figure legends

Supplemental Fig. S1.

Supplemental Fig. S2.

Supplemental Fig. S3.

Supplemental Fig. S4.

Table s1

Table s2 Primers

## Data Availability

The data sets used and/or analyzed during the current study are available from the corresponding author on reasonable request. RNA-sequencing data that support the findings of this study have been deposited in the NCBI BioProject repository (http://www.ncbi.nlm.nih.gov/bioproject/) with accession numbers PRJNA705039 and SRP224538.
