# Supplementary material for: ATF3 deficiency impairs the proliferative–secretory phase transition and decidualization in RIF patients
Source: Cell Death Dis. 2021 Apr 12;12(4):387. doi: 10.1038/s41419-021-03679-8 (PMC8041902; doi:10.1038/s41419-021-03679-8)
Supplement: Supplementary file 1 — Supplemental Figure legends [file 41419_2021_3679_MOESM1_ESM.docx]

**Supplemental Figure legends.**

Supplemental Fig. S1.

A. ATF3 mRNA levels were measured in different tissues/cells in humans using Affymetrix microarrays (data available from the Genevestigator database, https://genevestigator.com). The red arrow highlights the expression level in the placenta, uterus and endometrium. The placenta exhibits a high level of ATF3 expression, whereas its level ranges from medium to high in the uterus and endometrium. Data were generated with Genevestigator (Hruz T, Laule O, Szabo G, Wessendorp F, Bleuler S, Oertle L, Widmayer P, Gruissem W and P Zimmermann (2008) Genevestigator V3: a reference expression database for the meta-analysis of transcriptomes. Advances in Bioinformatics 2008, 420747).

B. RNA-seq analysis of the expression of ATF/CREB families in the endometrium of RIF patients and FER controls.

Supplemental Fig. S2.

The expression pattern of ATF/CREB families in hESCs treated with 0.5 mM 8Br-cAMP and 1 μM MPA (M+A) for different periods of time (0, 0.5, 1, 2, 4, 8, or 16 h) was evaluated by qRT-PCR.

Supplemental Fig. S3. ATF3 overexpression promotes decidualization of human endometrial stromal cells in vitro.

A. Correlation between ATF3 and PRL expression levels in endometrial biopsies (R2=0.6146, P = 0.0025).

B, C. Overexpression of ATF3 in hESCs could induce the expression and secretion of PRL. ∗ P < 0.05, ∗∗ P < 0.01 compared with the ATF3 0 moi group.

D. Immunofluorescence was performed to analyze the morphological transformation of hESCs.

Supplemental Fig. S4.

A. Heat map showing hierarchical clustering of gene expression in the control and ATF3 overexpression groups.

B. KEGG pathway enrichment analysis and GO analysis of the DEGs.

C. Knockdown of ATF3 with siATF3 in hESCs did not influence the expression of FOXO1 during *in vitro* decidualization. ∗∗∗ P < 0.001.

D. ATF3 overexpression altered the expression of cell cycle-related genes.

E. TC-seq analysis of the dynamic expression of genes regulated by siATF3 during *in vitro* decidualization.
