# Supplementary figures and images for: ATF3 deficiency impairs the proliferative–secretory phase transition and decidualization in RIF patients
Source: Cell Death Dis. 2021 Apr 12;12(4):387. doi: 10.1038/s41419-021-03679-8 (PMC8041902; doi:10.1038/s41419-021-03679-8)

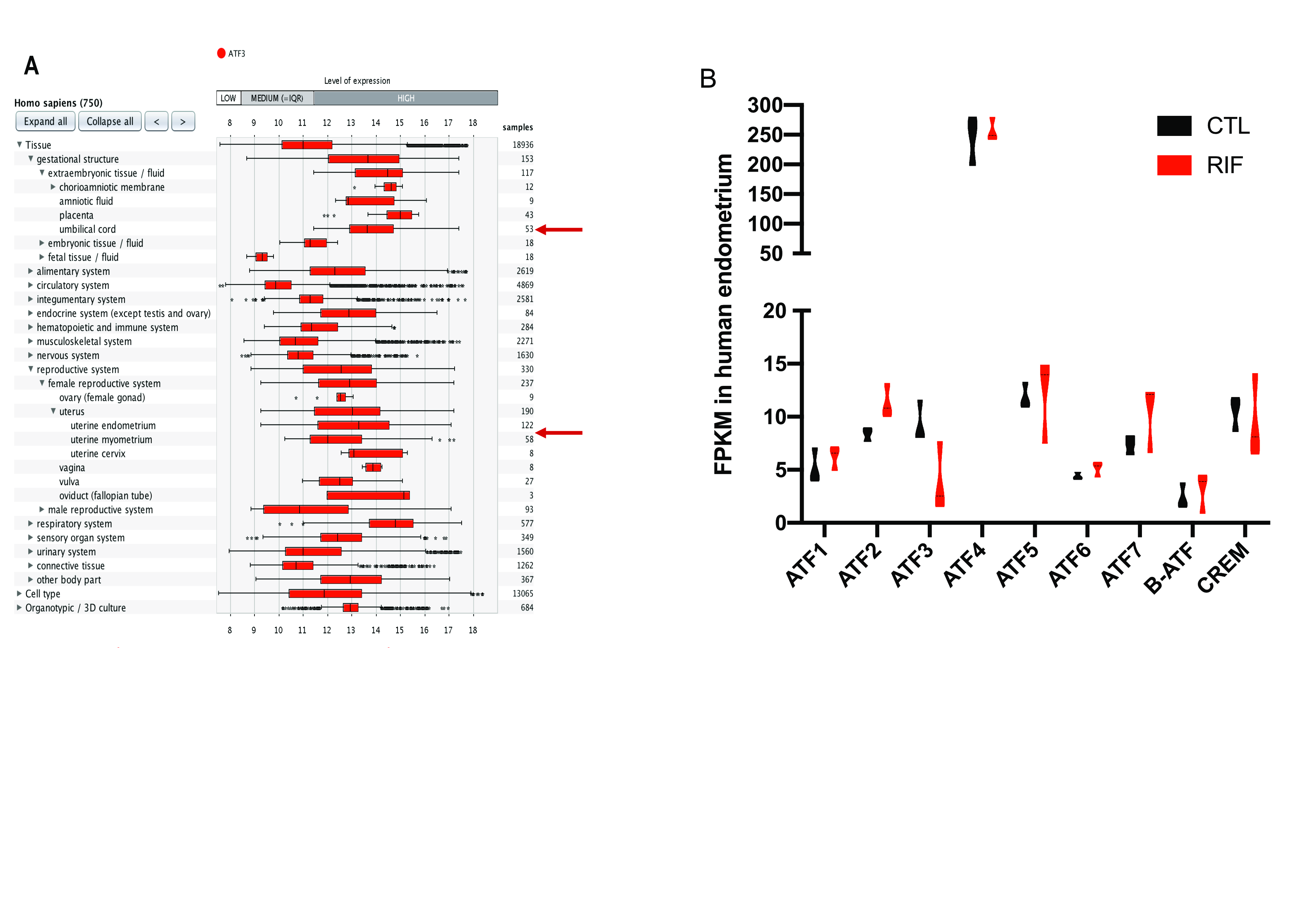

Supplement: Supplementary file 2 — Supplemental Fig. S1. [file 41419_2021_3679_MOESM2_ESM.tif]

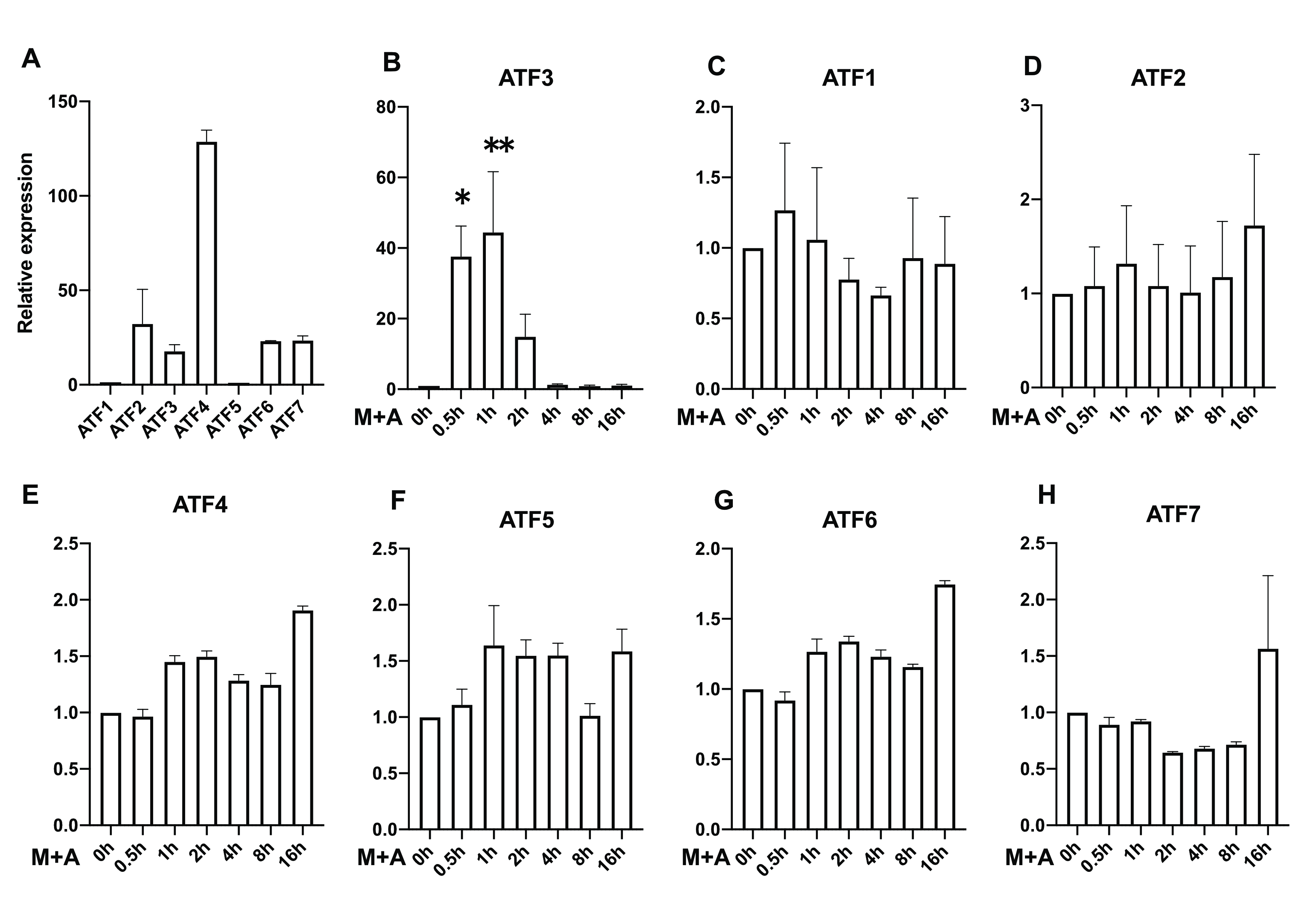

Supplement: Supplementary file 3 — Supplemental Fig. S2. [file 41419_2021_3679_MOESM3_ESM.tif]

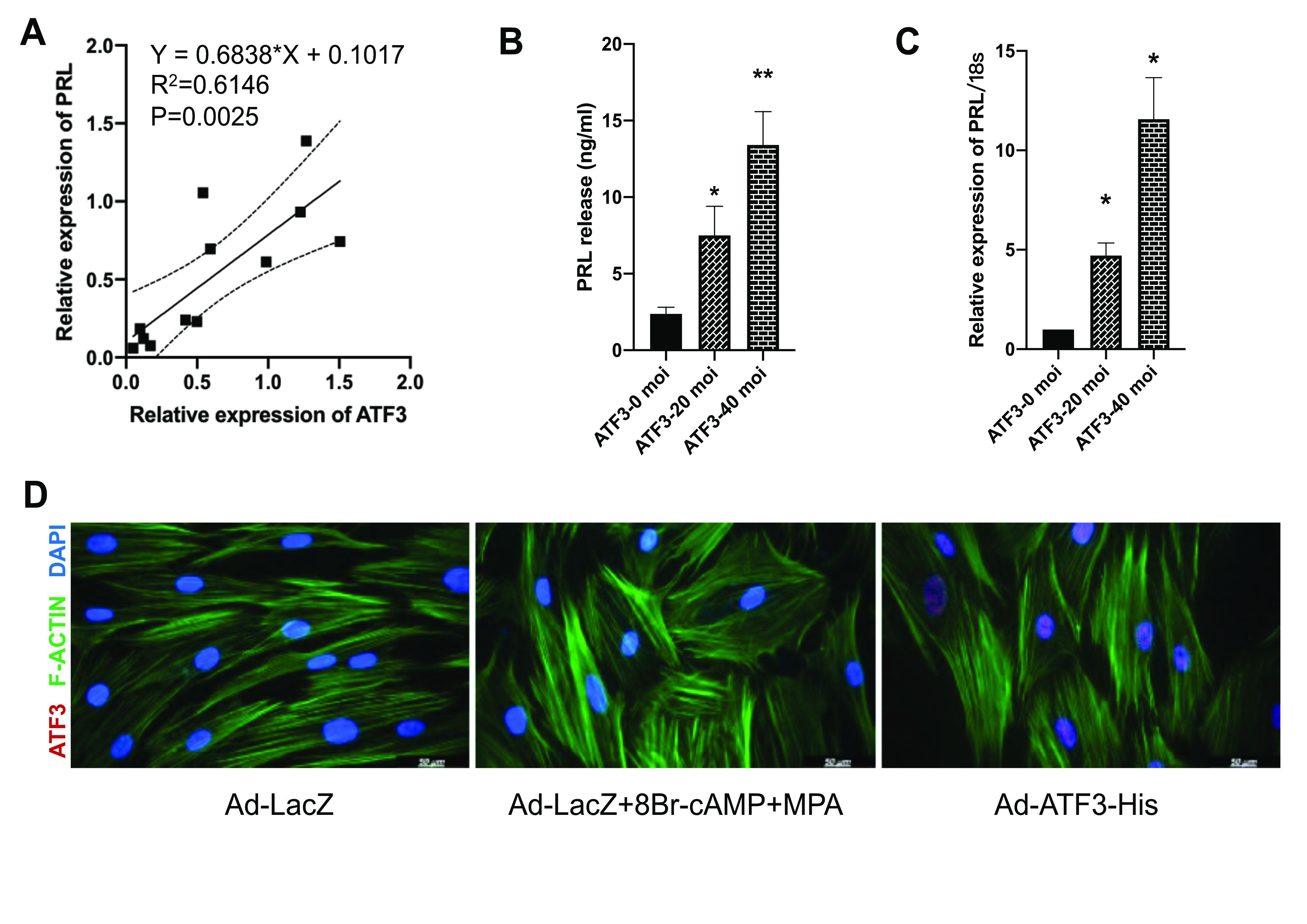

Supplement: Supplementary file 4 — Supplemental Fig. S3. [file 41419_2021_3679_MOESM4_ESM.tif]

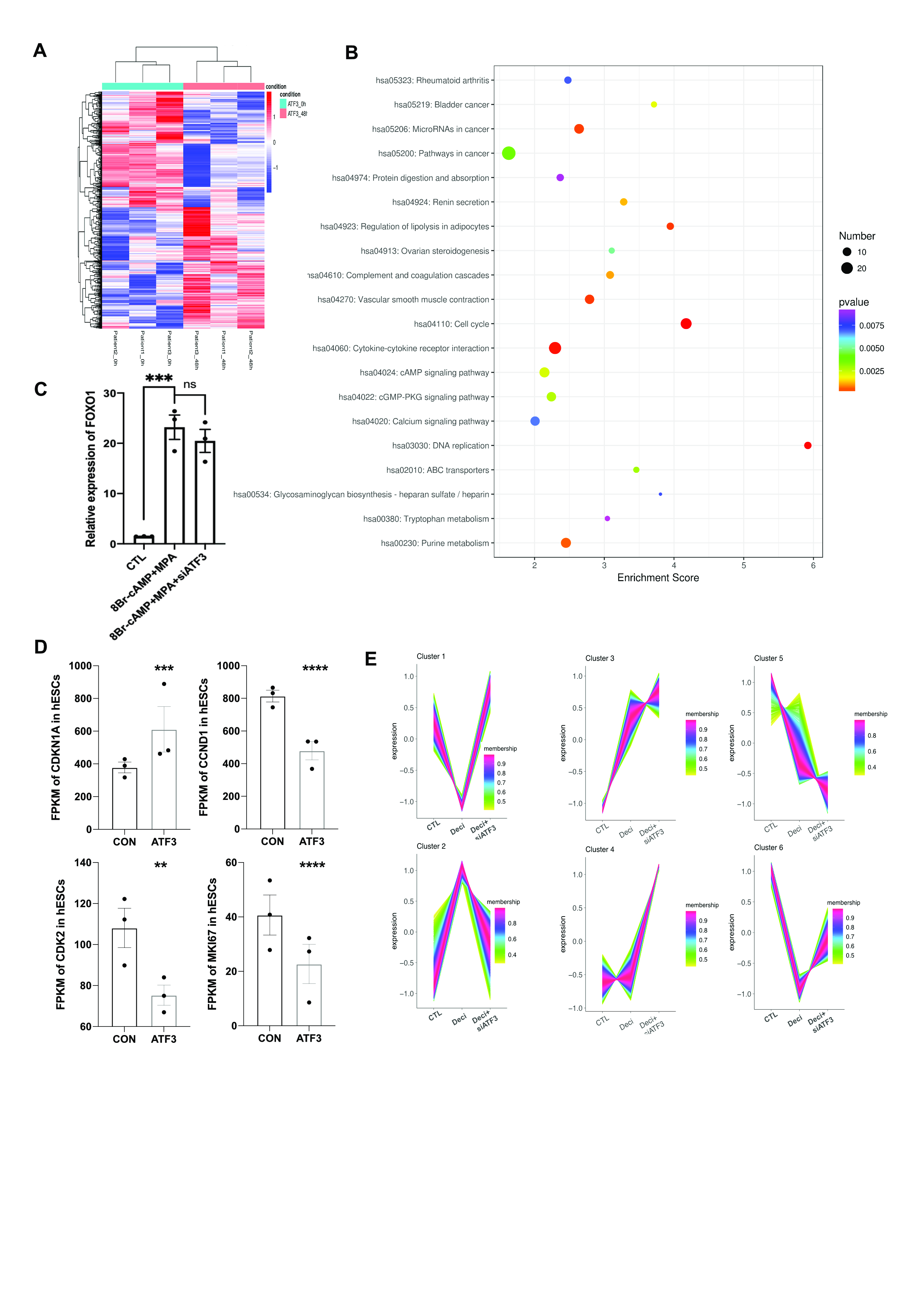

Supplement: Supplementary file 5 — Supplemental Fig. S4. [file 41419_2021_3679_MOESM5_ESM.tif]
