# Supplementary material for: ATF3 deficiency impairs the proliferative–secretory phase transition and decidualization in RIF patients
Source: Cell Death Dis. 2021 Apr 12;12(4):387. doi: 10.1038/s41419-021-03679-8 (PMC8041902; doi:10.1038/s41419-021-03679-8)
Supplement: Supplementary file 6 — Table s1 [file 41419_2021_3679_MOESM6_ESM.pdf]

	FER (n=51)	RIF (n=51)	p value
year	29.10±3.946	30.37±3.262	ns
BMI	21.81±2.396	21.39±2.660	ns
number of embryo transplantation	2.347±1.071	9.667±4.824	<0.0001
