# Supplementary material for: ATF3 deficiency impairs the proliferative–secretory phase transition and decidualization in RIF patients
Source: Cell Death Dis. 2021 Apr 12;12(4):387. doi: 10.1038/s41419-021-03679-8 (PMC8041902; doi:10.1038/s41419-021-03679-8)
Supplement: Supplementary file 7 — Table s2 Primers [file 41419_2021_3679_MOESM7_ESM.pdf]

Primer	sequence
h18S rRNA-F	CGGCTACCACATCCAAGGAA
h18S rRNA-R	CTGGAATTACCGCGGCT
hATF3-F	TCGGAGAAGCTGGAAAGTGT
hATF3-R	TCTGGAGTCCTCCCATTCTG
hATF1-F	CCAGCAATCAGGTGGTCGTACA
hATF1-R	GAGGTGAGAGTCACAGGAGATG
hdPRL-F	CACTACATCCATAACCTCTC
hdPRL-R	ATGCTGACTATCAAGCTCAG
hFOXO1-F	TCATGTCAACCTATGGCAG
hFOXO1-R	CATGGTGCTTACCGTGTG
has-miR-135b-F	ACACTCCAGCTGGGTATGGCTTTTCATTCCT
has-miR-135b-R	GGTGTCGTGGAGTCGGCAATTCAGTTGAG
U6-F	CTCGCTTCGGCAGCACA
U6-R	AACGCTTCACGAATTTGCGT
hIGFBP-F	TATGATGGCTCGAAGGCTCTC
hIGFBP-R	GTAGACGCACCAGCAGAGTC
h-PGR-F	GTCGCCTTAGAAAGTGCTGTCAG
h-PGR-R	GCTTGGCTTTCATTTGGAACGCC
h-CDKN1A-F	AGGTGGACCTGGAGACTCTCAG
h-CDKN1A-R	TCCTCTTGGAGAAGATCAGCCG
h-CCND1-F	TCTACACCGACAACTCCATCCG
h-CCND1-R	TCTGGCATTTTGGAGAGGAAGTG
h-DCN-F	GCTCTCCTACATCCGCATTGCT
h-DCN-R	GTCCTTTCAGGCTAGCTGCATC
h-FGF1-R	ATGGCACAGTGGATGGGACAAG
h-FGF1-R	TAAAAGCCCGTCGGTGTCCATG
h-BMP2-F	TGTATCGCAGGCACTCAGGTCA
h-BMP2-R	CCACTCGTTTCTGGTAGTTCTTC
h-TP53-F	CCTCAGCATCTTATCCGAGTGG
h-TP53-R	TGGATGGTGGTACAGTCAGAGC
h-WNT4-F	GCTGGAGAAGTGCGGCTGTGA
h-WNT4-R	CCACAAACGACTGTGAGAAGGC
h-PTEN-F	TGAGTTCCCTCAGCCGTTACCT
h-PTEN-R	GAGGTTTCCTCTGGTCCTGGTA
h-MAOA-F	TCTGAGCCTCACGAAGTGTCTG
h-MAOA-R	ATCCGTTCGCTCACTTGACCAG
h-TIMP3-F	TACCGAGGCTTCACCAAGATGC
h-TIMP3-R	CATCTTGCCATCATAGACGCGAC
h-IGF1-F	CTCTTCAGTTCGTGTGTGGAGAC
h-IGF1-R	CAGCCTCCTTAGATCACAGCTC
h-CDK1-F	GGAAACCAGGAAGCCTAGCATC
h-CDK1-R	GGATGATTCAGTGCCATTTTGCC
h-NOTCH-F	GGTGAACTGCTCTGAGGAGATC
h-NOTCH-R	GGATTGCAGTCGTCCACGTTGA
h-IHH-F	GGACGCTATGAAGGCAAGATCG
h-IHH-R	CAGCGAGTTCAGGCGGTCCTT

h-HAND2-F	GGCAGAGATCAAGAAGACCGAC
h-HAND2-R	CGGCCTTTGGTTTTCTTGTCGTT
h-NR2F2-F	TGCACGTTGACTCAGCCGAGTA
h-NR2F2-R	AAGCACACTGAGACTTTTCCTGC
